# Identifying dissemination strategies for promoting adoption of digital health interventions in clinical settings: A convergent parallel study on text message support for HIV pre-exposure prophylaxis (PrEP) adherence

**DOI:** 10.1371/journal.pdig.0001117

**Published:** 2026-07-13

**Authors:** Erik M. S. Ocean, Sonal D. Sharma, Tyler B. Wray

**Affiliations:** Department of Behavioral and Social Sciences, Center for Alcohol and Addiction Studies, Brown University School of Public Health, Providence, Rhode Island, United States of America; The University of British Columbia, CANADA

## Abstract

Many patient-focused digital health products that would be ideal for clinic-based promotion fail to gain traction in real-world practice, even when there is strong evidence that they improve health outcomes. Promoting adoption among both providers and patients in these settings remains a major challenge. For example, a recent U.S. Preventive Services Task Force review found that digital interventions such as smartphone apps and text-messaging programs increased the proportion of people taking PrEP as prescribed by about 10% compared with standard care. Despite these promising results, such interventions have seen little real-world uptake, and few studies have identified strategies to effectively promote their adoption in clinical settings or among patients. We conducted a convergent parallel, mixed methods study to identify a range of potential strategies for promoting adoption of a conceptual digital health intervention in medical outpatient clinics. Executives and leaders of PrEP clinics (*N* = 8) and established PrEP patients (*N* = 8) completed an in-depth qualitative interview followed by a survey. Interview data were analyzed using thematic analysis methods and survey data were analyzed using basic descriptive statistics. Qualitative findings identified main themes via executive interviews and patient interviews, with much overlap. Clinic executives and patients perceived direct, trusted communication from providers as the most likely driver of adoption for digital health interventions. Both groups also highlighted the potential roles non-clinic staff play in providing additional details. Additionally, both groups identified in-clinic and out-of-clinic general marketing strategies. Quantitative survey results generally confirmed these findings, with patients rating direct mentions from providers as a strategy most likely to lead to adoption, and providers rating discussions in provider meetings and direct recommendations from colleagues among the most likely to encourage them to recommend it to patients. Together, these findings identify promising dissemination strategies that should be tested in future studies.

## Author summary

Many patient-focused digital health tools that would be ideal for clinic-based promotion fail to be used in the real-world, even when there is strong evidence that they improve patients’ health. Promoting adoption among both providers and patients in these settings remains a major challenge. In our study, we interviewed clinic executive leaderships and patients separately to identify strategies to effectively promote their adoption in clinical settings, among patients. We considered the workload of providers and their staff, the relationships that providers have built with their patients, and patients’ views on products marketed towards them, both inside and outside their clinic. Results showed that a direct provider-to-patient recommendation is the most likely driver of adoption of a digital health tool. We acknowledge that providers have limited time to spend with each patient and highlight that potential role that non-clinical staff can have in filling that gap. Last, our participants helped identify general marketing strategies to be deployed in- and out-of-clinic as well.

## Introduction

Rates of HIV in the United States (US) are declining, but not rapidly enough to meet the Ending the Epidemic Initiative’s goal of a 90% reduction in new infections by 2030 [1]. Nearly 70% of all new infections continue to be diagnosed among men who have sex with men (MSM) and transgender women (TGW), far and away the most-affected populations in the US [2]. Nearly half of those newly diagnosed with HIV live in the US South [2]. Improving coverage and use of HIV pre-exposure prophylaxis (PrEP) medications is one step that can help achieve more pronounced, sustained declines in new infections [3,4], but current use is too low to meet these goals [5]. Long-acting, injectable forms of PrEP like lenacapavir and cabotegravir, have enormous promise for achieving more optimal coverage of exposures while minimizing adherence burden. However, these medications are still in the early stages of rollout, access is variable, and at least some evidence suggests a significant percentage of those who are most at-risk may still prefer daily pills [6]. Finding ways to promote optimal adherence to any PrEP product will continue to be a challenge for the foreseeable future. A recent Community Preventive Services Task Force systematic review found that digital health interventions such as text messaging and smartphone apps increased PrEP adherence by a median of about 10 percentage points for ‘good adherence’ (≥4 doses/week) and 20 percentage points for ‘excellent adherence’ (7 doses/week) compared with standard care [7]. In the years since this review was published, however, none of the included interventions have moved meaningfully beyond the research setting, with very limited use reported outside of research settings.

For digital health products like these, clinics are uniquely positioned to identify patients who could benefit from them (e.g., individuals prescribed PrEP) and to provide trusted guidance on their use. That is, since providers regularly interact with these patients and are respected sources of health information, clinics are particularly well suited to promote adoption of these tools. Clinics also have a natural alignment of interests for promotion of these products, as they have been proven effective in improving patient outcomes. Adoption in clinical settings, however, hinges on multiple factors, including the strength of evidence supporting the product’s health benefits and stakeholders’ perceptions of both those benefits and the tool’s overall utility [8]. Features of the tool itself also play a role. Factors like the time and effort required for providers to refer patients, the degree of workflow disruption, and how the tool is prioritized relative to other tasks influence uptake [8]. Tools that are not designed to integrate seamlessly into clinical workflows allowing providers to refer patients with minimal effort, are unlikely to achieve widespread adoption. Critically, real‑world uptake has also been limited partly because the strategies that most effectively drive adoption within medical clinics remain poorly understood. Evidence is scarce regarding which approaches best secure leadership buy‑in, what motivates providers to recommend these tools, and what other approaches might reach patients. This problem extends beyond PrEP-focused solutions: many digital health interventions are suitable for clinic-based dissemination but confront similar adoption barriers.

The limited available literature suggests that patients in clinical settings may be most likely to adopt digital health tools when outreach is personal and convenient. A systematic review of dissemination strategies for digital health tools reported that face‑to‑face promotion and general marketing methods were among the most commonly-reported strategies used in past research [9]. For example, some were promoted via physicians or practice nurses [10,11]. Others were promoted via physical signage, social media ads, or via email [12–14]. However, only one of the included studies reported data about the effectiveness of the approaches used. A qualitative study of an asthma and allergy app showed that social media ads encouraged many downloads but yielded low engagement, while direct referrals from practice nurses encouraged fewer downloads but stronger engagement [11]. Qualitative research with patients supports these findings, showing that patients believed that having providers discuss the product at opportune moments during clinics visits could improve adoption, in addition to global marketing efforts to ensure awareness [15]. Together, these studies suggest that, while basic marketing approaches like physical signage and direct emails are necessary, encouraging providers to discuss the product directly with their patients could be an especially effective strategy.

What strategies could encourage healthcare clinicians to talk to their patients about adopting a digital health product? An umbrella review found that ensuring providers were aware of the product, promoting its usefulness, providing any training (if necessary), and appointing local champions were among the most common facilitators of digital health adoption among clinicians [8]. Champions are individuals who are embedded in a healthcare organization whose job is to promote a new technology and help colleagues integrate it into routine care. Champions discuss tools with other staff members (e.g., presenting at staff meetings, organizing group demos, meeting with staff one-on-one) and support them as they use it (e.g., answering questions over the phone and email) [16–18]. Although not focused on adoption of digital health specifically, a study that used electronic medical record (EMR) message to encourage providers to counsel patients with a positive sexually-transmitted infection test to consider PrEP increased the odds that providers documented having discussed PrEP with patients [19]. This finding suggests that EMR alerts could be an effective strategy for encouraging providers to discuss adopting a digital health tool with their patients. Other studies have designed campaigns involving many strategies to engage providers. For example, a study of a “multi-channel” effort found that email outreach, WhatsApp messages, in-person training, and video presentations were effective in encouraging healthcare providers to use an app that screens patients for skin conditions [20]. Similar studies that encouraged clinics to use multiple channels based on local needs also show promise [21]. Together, these studies suggest that strategies that combine champions who engage colleagues directly (e.g., presenting at staff meetings) with multi-channel outreach to raise awareness of the product (e.g., signage, email) could be especially promising.

We conducted a convergent parallel mixed methods study with PrEP clinic executives and established PrEP patients to identify strategies that they thought might be most effective for disseminating a conceptual digital health product designed to support PrEP adherence. Interview questions were influenced by Brownson et. al.’s conceptual dissemination framework which focuses on approaches for active diffusion of innovations [22]. Brownson et. al. describe effective dissemination as tailored, audience specific, and using multiple channels. Both groups were asked to suggest dissemination strategies they believed might be most effective in encouraging patient adoption. Among clinic executives we probed organizational fit, workflow impact, and burden. With patients, interviews focused on communication fit and trust. Participants in both groups completed an in-depth, qualitative interview, followed by an online survey. We then compared and contrasted results from both methods. We selected this approach so that we could use qualitative interviews to surface a wide range of potential strategies and elicit *why* stakeholders thought particular approaches would work, which could be helpful for researchers selecting from among various available strategies. The quantitative work then allowed us to examine whether participants’ rationales aligned with their ranked preferences. This understanding is valuable not only for identifying specific strategies that can be empirically tested in future studies, but also for ensuring that dissemination efforts are grounded in the perspectives and practical experiences of both clinic decision-makers and patients.

## Methods

### Ethics statement

Procedures were approved by the Brown University Institutional Review Board (ID: STUDY00000600). Participants provided formal informed consent electronically before beginning the study.

### Participants

We recruited executives of PrEP clinics (*N* = 8) in the US South via email or direct messages on LinkedIn. Executives were eligible if they (1) were working in a leadership role in a clinic that currently prescribed PrEP to patients, (2) made decisions about service delivery or programming at their clinic, and (3) their clinic was operating in a state in the US South (e.g., Alabama, Georgia, Louisiana, Mississippi - see S1 Appendix for a full list). PrEP patients were recruited from a registry of individuals in the US South who had expressed interest in engaging in research. Patients were eligible if they (1) were 18 + years old, (2) assigned male sex at birth, (3) reported sex with a man in the past 12 months, (4) speak and read English fluently, (5) reside in a state in the US South, (6) were prescribed PrEP, (7) reported actively taking PrEP, and (8) had access to a device they could use to participate in a videoconference meeting. These criteria were inclusive of both men and transgender women (TGW). We elected to focus on men who have sex with men (MSM) and TGW, given that these populations account for nearly 70% of all new HIV infections in the United States [2]. Both groups were included because there was no clear scientific rationale for focusing exclusively on one group or the other, and we believed both could provide helpful insights about the best ways to reach them about digital health tools that could be helpful for them.

### Procedures

Patients who met eligibility criteria were asked to provide informed consent online before scheduling an appointment for their interviews using an online scheduling system. Clinic executives who expressed interest in participating were provided a link to schedule an appointment to complete their interviews. Clinic executives were not formally required to provide informed consent because they were considered “key informants” who were solely being asked to provide their professional perspectives on an issue relevant to their work, and thus, were not considered human subjects. However, interviewers covered basic elements of informed consent prior to beginning each interview with both executives and patients and offered to answer any questions. All interviews were conducted via videoconferencing, were recorded for later transcription, and lasted up to 90 minutes. They were conducted by trained and experienced research personnel with advanced degrees (all authors). After interviews were completed, participants were sent a follow-up survey via email. All interviews were conducted between December 2024 and April 2025. Executives were paid $200 for completing both portions and patients were paid $50. These procedures were reviewed and approved by the Brown University Institutional Review Board.

### Measures

#### Semi-structured interviews.

For executives, semi-structured interviews first asked interviewees to introduce themselves, their roles at their clinics, and to describe the size and populations of PrEP patients their clinics generally served. For patients, interviews first asked participants to describe their experience with PrEP. All participants were then asked about their perceptions of the most important barriers to encouraging more PrEP use and to supporting adherence. Then, to provide context for questions about dissemination strategies, interviewers described the characteristics of a prospective digital health intervention to support PrEP adherence. The prospective tool we described was based on text messaging adherence support interventions that have shown promising results in past studies [23,24]. We showed participants mockups of a website (S2 Appendix) that facilitated onboarding participants into the prospective program by showing them how one would submit only their phone number to register and then ask them to confirm via text message before continuing the program over text. We asked participants to assume that the program would be designed to onboard participants as quickly and easily as possible, with minimal effort from referrers, would be easy for patients to use, and that any marketing materials would be attractively designed. Participants in this study were not actually asked to onboard into the prospective program. We adopted this approach so that we could focus questions exclusively on participants’ views of potential dissemination strategies, rather than other concerns, such as the design characteristics of the intervention itself or the appearance of associated marketing materials. After presenting this information, in executive interviews, we asked interviewees whether they believed their clinic staff and providers would be interested in helping to promote a similar tool, before asking them to reflect generally on what promotional strategies they believed might be most effective in encouraging patient adoption. In patient interviews, we asked participants whether they believed other patients like them would be interested in using a similar tool, before asking them to reflect generally what promotion strategies they believed they might be most likely to (1) notice and (2) encourage them to sign up. Interviewers probed responses to these queries to encourage participants to explain why they believed the strategies they noted could be effective. Then, interviewers asked about several specific strategies that participants had not mentioned but that have appeared in the literature. For each strategy we inquired about, we asked follow-up questions addressing whether they thought each strategy would be effective, and if so, why. For executives, we also asked whether their clinics had opportunities to use each strategy and whether they would be supportive of each strategy. The additional strategies we asked about included general marketing methods of physical signage, physical handouts (e.g., postcards, palm cards), digital signage (e.g., ads on any TVs or monitors in the clinic), as well as digital marketing (e.g., social media posts or paid ads), direct emails or messages to patients, or clinic newsletters. In executive interviews, we also asked interviewees about various strategies for encouraging providers to refer their patients to the tool, such as hosting in-person trainings or informational meetings (e.g., presentations at staff meetings, demonstrations) or appointing local champions.

#### Follow-up survey.

For both groups, online follow-up surveys collected after interviews assessed basic demographics, including age, sex at birth, gender, race, and ethnicity. Patients were also asked several questions about their history and experience with PrEP. For executives, items then assessed the likelihood they would be to recommend that their clinic’s PrEP patients sign up for the program, that their colleagues refer patients to it, and that it be promoted clinic wide. General acceptability, feasibility, and appropriateness of the described intervention were assessed in both executives and patients using the Acceptability, Feasibility, and Appropriateness of Intervention Measures [25]. Each of these measures is four-items for a total of 12 items, each rated on a 1 (*completely disagree*) to 5 (*completely agree*) scale. These scales have shown substantive validity and acceptability reliability in past studies [26]. Reliabilities for each scale were excellent in this study, both among clinic executives (ɑ = 0.95, ɑ = 0.89, and ɑ = 0.89, respectively) and patients (ɑ = 0.96, ɑ = 0.89, and ɑ = 0.87). Executives were then asked to rate several potential dissemination strategies, most of which we identified from previous literature, in terms of which they believed would be most effective in capturing providers’ attention and encouraging them to recommend the digital health tool to their patients. Each strategy was rated on a 1 (*not likely at all*) to 5 (*very likely*) scale. Example items include “a flyer posted in the clinic where you work,” and “someone presenting about it in a special presentation at the clinic where you work” (see S3 Appendix for specific items). Patients were asked to rate similar items, except that they were first asked to rate which dissemination strategies they believed would make them most likely to *notice* information about the product and which they believed would be most likely to *encourage them to sign up* for the product. Each was rated on a similar scale from 1 (*not likely at all*) to 5 (*very likely*). Some additional potential strategies that would not necessarily involve clinics directly were included in patient surveys (e.g., “A flyer posted around the area you live,” “An email from your insurance company”) because, for these strategies, patients were best suited to judge whether they would be likely to notice or sign up in response to these approaches. Finally, to identify aspects of the intervention that could be emphasized in any future marketing efforts, executives were also asked to rate how important various aspects of the intervention or marketing approach would be to their decision to recommend or promote the tool. Example items include “how easy it is for patients to sign up,” and “its security,” with each rated on a 1 (*not at all important*) to 5 (*extremely important*) scale. Patients were similarly asked what barriers would be most important to their decision to sign up for the intervention or not. Example items included “I don’t have a phone or free texts” and “it doesn’t sound interesting to me,” with each rated on a 1 (*not at all a reason to not sign up*) to 5 (*very much a reason to not sign up*) scale.

### Data analysis

Results were analyzed using inductive qualitative thematic analysis methods in NVivo. A priori and de novo codes were used. A combination of essentialist and constructionist frameworks were implemented during analysis; we began coding transcripts focusing on description of topics discussed and progressed to interpretive codes as they were uncovered. Two authors (EMSO and SDS) independently coded the first three transcripts of each participant group (i.e., executives and patients) and met to review and finalize the codebook before continuing to independently code each transcript. Coders took notes during the coding of each transcript, recording decisions, reasoning, challenges, shifting assumptions, and code drift. Coders collaborated reflexively, relying on each other to share differing perspectives after independently coding transcripts. The full team met regularly to discuss notes, coding, and findings, actively auditing their own and the other coder’s analytical decisions. Codes in each transcript were systematically resolved. Special attention was given to inductive thematic saturation, with ongoing monitoring of the coding process as no new codes or themes emerged. Upon agreement of final coding, authors identified codes of interest, developed themes, and selected representative quotes. Authors did not edit the language of the quotes, except to remove pauses, word fragments, fillers, and hesitation markers for clarity.

## Results

### Demographics

Participant demographic and summary characteristics for clinic executives are presented in Table 1 and for patients in Table 2. Clinic executives largely led federally qualified health centers and other non-profits and hospital-affiliated clinics, with the median clinic serving around 600 PrEP patients. Patients were generally well-educated and employed full-time with extensive (4 + years) of PrEP experience and few challenges adhering to their medication.

**Table 1:** Demographic characteristics of clinic executives (*N* = 8).

Characteristic	Clinic executives (*N* = 8)
	N (%) or *M* (*SD*)
Age (34–70)	51.4 (13.65)
Gender	
Woman	4 (50.0)
Man	3 (37.5)
Transgender woman	1 (12.5)
Hispanic or Latine ethnicity	0 (0.0)
Race	
White	5 (62.5)
Black or African American	3 (37.5)
Doctoral degree	3 (37.5)
Type of PrEP Clinic	
University/hospital affiliation	2 (25.0)
Health department affiliation	0 (0.0)
Federally qualified health center	3 (37.5)
Other not-for-profit	3 (37.5)
Private	0 (0.0)
Estimated Number of PrEP Patients	
200-499	3 (37.5)
500-999	2 (25.0)
1000-1999	2 (25.0)
2000+	1 (12.5)

**Table 2:** Demographic characteristics of PrEP patients (*N* = 8).

Characteristic	PrEP patients (*N* = 8)
	N (%) or *M* (*SD*)
Age (24–52)	35.9 (9.6)
Sex at birth	
Female	0 (0.0)
Male	8 (100.0)
Neither	0 (0.0)
Gender	
Woman	0 (0.0)
Man	8 (100.0)
Transgender woman	0 (0.0)
Transgender man	0 (0.0)
Other, non-binary, genderfluid	0 (0.0)
Hispanic or Latine ethnicity	3 (37.5)
Race	
White	6 (75.0)
Black or African American	2 (25.0)
American Indian or Alaska Native	0 (0.0)
Asian (including the Philippine and Indian Islands)	0 (0.0)
Pacific Islander (including Hawaii)	0 (0.0)
College degree	8 (100.0)
Unemployed	0 (0.0)
Low income^1^	1 (12.5)
Gay or bisexual	7 (87.5)
PrEP medications currently prescribed	
Emtricitabine/tenofovir disoproxil fumarate	4 (50.0)
Emtricitabine and tenofovir alafenamide	4 (50.0)
Cabotegravir	0 (0.)
Provider who currently prescribes PrEP	
PrEP, HIV, or STD clinic	2 (25.0)
Primary care provider	4 (50.0)
Telehealth company (e.g., Mistr, QCare+)	2 (25.0)
Pharmacist	0 (0.0)
Other	0 (0.0)
Average time on PrEP, in years	4.7 (3.1)
# days adherent to PrEP, past 30 days	29.6 (0.7)
Difficulty taking/getting PrEP on schedule^2^	1.25 (0.5)

^1^Low income = annual income of <$40,000. ^2^On a scale of 1 (*not at all difficult*) to 5 (*extremely difficult*).

### Qualitative interviews with clinic executives

Clinic executives’ reactions to the digital health product concept were positive, with many suggesting that such a tool could meet an important patient need and seemed feasible to promote in clinical settings. Queries about potential dissemination strategies yielded four main themes.

#### Theme 1: Direct provider-to-patient recommendation, staff follow-up.

Clinic executives consistently emphasized the importance of providers in encouraging patients to adopt a digital health PrEP adherence support program. Several noted that provider referrals could be especially powerful because the providers’ endorsement holds significant weight, given that patients value their advice and guidance:

*“We do know that patients [...] put stock into what their provider tells them. So I feel like it is important for the provider to say something about it.” – Clinic executive #2*

However, many clinicians also acknowledged the reality of the time pressure that providers are under. They noted that providers are often limited to 15 minute visits with participants, which often means that a single visit does not provide them with enough time to discuss everything. Though, some also acknowledged that having a simple tool that they could point patients to could be a valuable way to address adherence during these brief appointments:

*“I’m always under time pressure. I want to do something to address adherence. Once I open up that can of worms, you know, a visit for which I had 15 minutes can turn into a 40 minute visit. If I can just point to something and say, hey, these people are really expert in helping you stay on your medications.” – Clinic executive #8*

One solution executives offered to address the limitations on providers’ time was for providers to recommend that patients consider signing up for the tool, with other clinical and support staff following up afterward to provide more information and any training necessary to sign up. They suggested that having a designated individual such as a nurse or patient navigator, who can offer more in-depth information, answer patient questions, and provide comprehensive details is key.

*“If the promotion… is promoted by every person that the person interacts with… I also think that would bring home the point and the message.” – Clinic executives #4*

#### Theme 2: In-clinic physical signage, with QR codes.

Clinic leaders also suggested that making physical signage and handouts (e.g., postcards, palm cards) available in clinics could be another way for clinicians to save time during appointments. This way, providers could quickly suggest the product to their patients who are on PrEP and then provide them with a handout they can use to learn more about it and sign up, if interested. Executives also specifically emphasized that including a QR code on physical signage and handouts could ensure that patients can easily scan them to connect to an onboarding website:

*“Sometimes it’s best to outsource. And if you make the outsourcing as easy as telling the patient… scan this QR code… they’re going to link you to a whole bunch of resources… that would be awesome. And it doesn’t take very long to do that. [...] If all you have to do is point to a QR code and say, scan this, it will do my job for me.” – Clinic executive #8*

#### Theme 3: Post-visit communications - Patient portal messages, appointment summaries, emails, texts.

Executives also identified several other communication channels that could be promising for promoting a digital health tool beyond providers’ discussions during visits. They suggested that sending emails or text messages to patients who were newer to PrEP could be another strategy to increase awareness of the intervention, although clinics directly contacting patients in this way is relatively uncommon. Messages to patients sent through their patient portals was another strategy participants raised that they believe would be consistent with typical practice, but not all patients engage with portals. Finally, executives also suggested that including information about the program in patients’ appointment summaries could provide another physical reminder of the program:

*Having a pamphlet or having… a way to get into the after visit summary… have the provider give the patient… would be keen.” – Clinic executive #7*

#### Theme 4: Digital marketing.

Executives also suggested that outside advertisements could be effective, but would require a strategic approach. Many related that their own experience with traditional advertising methods like billboards, radio, and television ads were not effective in engaging their target demographic, which often skewed younger since risk for HIV and sexually-transmitted diseases are more prevalent among younger age groups. Many believed that social media would be a more promising approach for engaging these populations:

*“I think for the younger populations… in our data from 2022, the numbers of new diagnoses for HIV, syphilis, chlamydia, gonorrhea were in the 20 to 29 year old age groups, right? So they’re using social media. They’re not listening to the radio. They’re streaming, maybe some streaming type stuff they’ll listen to. But Instagram, social media, that’s where they’re going to get their information… And boosting posts is very cheap” – Clinic executive #3*

### Clinic executive survey results

Survey data showed that executives had very positive views of the concept PrEP support tool. On average, they reported being likely to recommend that their clinic’s PrEP patients sign up for such a product (*M* = 4.63, *SD* = 0.52, Range = 4–5), to recommend that their colleagues refer their patients to it (*M* = 4.75, *SD* = 0.46, Range = 4–5), and that their clinics promote it clinic-wide (*M* = 4.63, *SD* = 0.52, Range = 4–5). They also gave the concept high ratings of acceptability (average item *M* = 4.22, *SD* = 0.41, Range = 4–5), feasibility (average item *M* = 4.08, *SD* = 0.46, Range = 3–5), and appropriateness (average item *M* = 4.17, *SD* = 0.36, Range = 4–5). Table 3 shows ratings of how effective they believed various dissemination strategies would be in making providers and clinic leaders aware of a digital PrEP support tool and recommend it to patients. Strategies like having someone present about it, either at their clinics’ regular staff meetings or in a special presentation, and having a colleague promote it, either by talking to other providers about it or distributing promotional materials, were among the most highly rated. Placing handouts in locations around the clinic that providers frequent (e.g., conference rooms) was the least highly rated, although even this strategy was mostly rated ‘neutral.’ Table 4 shows ratings of how important various characteristics of the tool might be in their decision to promote a tool like the one described. These ratings could be useful in informing what aspects of the product might be emphasized in marketing materials to be most effective. Ease of signup for participants, the strength of the evidence supporting the clinical benefits of using the tool, cost, and ease of provider referrals were among the most highly rated characteristics, with testimonials from other providers and patients receiving the lowest ratings. However, again, executives still rated even the lowest-rated characteristics as “somewhat important.”

**Table 3:** Clinic executives’ ratings of how likely various dissemination strategies might be in making providers aware of a digital PrEP support tool.

Strategy	*M*	*SD*
Someone presenting about it at your clinic’s regular staff meeting	4.75	0.46
Someone presenting about it in a special presentation at your clinic	4.75	0.46
A colleague who works at your clinic directly mentioning it to you	4.75	0.46
A colleague who works at your clinic handing out or wearing promotional materials, like buttons, pens, or information cards	4.38	0.52
An ad on a TV or screen in your clinic	4.38	0.74
A message sent to you in your electronic health records system that your clinic uses	4.25	0.71
A text message sent directly to you	4.25	1.16
A flyer posted in your clinic	3.75	1.16
A headline about it in the email newsletter of your clinic	3.75	1.16
Business cards placed on tables in common areas (e.g., conference room) at your clinic	2.88	1.13

**Table 4:** Clinic executives’ ratings of how important various aspects of a digital PrEP support tool would be to their decision to promote it to providers and recommend it to patients.

Characteristic	*M*	*SD*
How easy it is for patients to sign up for it	5.00	0.00
Research evidence showing that it encourages more consistent use of PrEP	4.88	0.35
Its cost	4.75	0.46
How easy it is for providers to refer people to it	4.75	0.46
Its security	4.63	0.52
Whether it addresses a specific topic that contributes to poor adherence, like stigma or risk perceptions	4.5	0.76
Its promotional materials (website, ads, flyers) look professional	4.5	0.76
Testimonials from other providers saying it helped their patients	4.13	1.13
Testimonials from patients saying it was helpful for them	4.00	1.07

### Qualitative interviews with patients

Patient reactions to the adherence support program concept were also positive, with many suggesting that such a tool could be helpful for other patients like them. However, the patients in this study reported taking PrEP for an average of 4.7 years, being highly adherence, and having low difficulty taking/getting PrEP on schedule (see Table 2). Several added that they believed a product like this would mostly be helpful for patients who were newer to PrEP than many of them were.

*“I’ve taken PrEP for seven years. So I would not want to use this product, but that has nothing to do with the product itself. That just comes from the fact that I’m very well versed on this. … If it was me seven years ago, I [...] absolutely would sign up for the product.” Patient #7*

Still, many noted that the concept seemed feasible to sign up for and use. Similar to clinic executives’ reactions, queries to patients about potential dissemination methods could be separated by in-clinic and external or ‘out-of-clinic’ strategies, shown in Table 5, weighted by both by prevalence of findings and strategic importance.

**Table 5:** Patients’ ratings of various barriers to adoption of a concept digital PrEP adherence support tool.

Characteristic	*M*	*SD*
I don’t think I need it	2.88	2.03
I don’t want to get tons more text messages	2.75	1.49
I don’t see how it would benefit me	2.25	1.75
I don’t think it would help me take my PrEP regularly	2.00	1.60
It doesn’t sound interesting to me	1.88	1.64
It seems like a scam	1.63	1.19
No one else I know has used it	1.50	0.76
I don’t want to go through the effort of signing up	1.38	0.74
I don’t have access to a phone or free text messaging	1.00	0.00

#### Theme 1: Direct provider-to-patient recommendation.

Patients generally agreed with executives that various efforts to promote a product like this might be most effective if the focus were on publicizing it *within* clinics. Patients consistently emphasized the strength of provider recommendations. Many specifically noted the high level of trust they had in their providers: *“I’ve been around my doctor long enough to know that [they] say, see things, most things in my best interest. So it’s like … I said, hey, I’ll check it out.” – Patient #5*

#### Theme 2: Post-visit communications - Patient portal messages, appointment summaries, emails.

Patients also believed that messages sent from clinics in various ways, such as email, text message, or patient portal notifications, could be effective, again because of the trust they have in these institutions. Several likened communications from these clinics to direct recommendations their providers might share with them, and suggested that the levels of credibility and trust were similar:

*“I think one thing that is huge about doing it that way is you already have kind of the trust of the provider or the clinic. So someone like [name of specific area clinic] that I previously got, as my healthcare clinic and got my PrEP through. If they were to reach out about a product, I think I would be much more likely to trust that product if it’s kind of coming with their recommendation or with their brand.” – Patient #1*

Although patients agreed that communication from their clinics could be uniquely influential, they differed in their views about *what type* of communication they might be most likely to attend to. Some suggested that strategies like emails and newsletters might be less effective because they tend to get lost among the many other promotional emails they receive, and that patient portal messages and appointment summaries might be particularly effective because messages they receive through the portal tend to be highly important and relevant. Others thought promoting via email would be useful.

*“I’m not sure what the impact would be and how much we would get out of [emails and newsletters]. Through the patient portal. Yes, I think that I would… whatever it’s on the patient portal is important; I pay attention to it. But now again, I only go there whenever I have a visit to my doctor. So, it’s not very frequently.” – Patient #8*

#### Theme 3: In-clinic physical & digital signage, handouts.

Patients also had mixed views about how effective physical signage posted around clinics might be. Several reported that they frequently review physical and digital signage around the clinics they attend, either because they enjoy them and have a habit of perusing them or because they serve as a distraction when undergoing various procedures.

*“I would [take notice of the advertisement in my PrEP clinic]. I’m old school I still like physical products so like if I did see a flyer and, you know, says, hey, check this out [...], then yes, I would definitely grab it and study it and QR code it, whatever. Yes, most definitely.” – Patient #4*

Other patients were less enthusiastic about these strategies, either because they prefer to use their phones when they have idle time or because they prefer directly interacting with people.

*“Probably not. Probably not, nowadays people, I think that we are glued to our phone. Even if you’re waiting and there are a zillion printed items, you’re not going to read them.[...]. I just go in, sign my name, go onto my phone.” – Patient #8*

*“I am very interactive. So if someone presents something to me, I’m going to be a lot more, I’m going to be a lot more likely to pay attention to it and pursue it than if it’s just being casually presented to me on TV or through a website.” – Patient #7*

Finally, other patients were skeptical of in-clinic signage as a dissemination approach because they use a telehealth service to access PrEP. In this sample, a sizable minority of participants were prescribed PrEP through such a service, so other approaches would be needed to reach these patients, such as purely web-based promotion.

#### Theme 4: Digital marketing.

In addition to the variety of in-clinic approaches raised, PrEP patients also discussed several potential strategies outside of clinics that they believed could be effective. Most patients believed that various online platforms would be effective ways to reach many PrEP patients to promote digital health tools, but there was disagreement about how effective specific online marketing strategies might be. Although some mentioned that social media ads could be useful, many reported paying only occasional attention to them.

*“I actually do get some just general information with HIV drugs coming through on my Facebook and stuff. I am on Facebook and Instagram a lot so actually, Facebook and Instagram, I would take notice.” – Patient #7*

Others emphasized that using influencers popular with priority audiences could be powerful, since their messages are more likely to be memorable and prompt viewers to engage.

*“There is a drag queen that does an advertisement for some healthcare service … So that is memorable to me in the fact that I remember that now makes me think that if there were an influencer or someone who is more famous who’s promoting this, I would at least remember it, recall it, or be likely to look into it, yeah.” – Patient #1*

However, others had strong reservations about using influencers, noting that they often seem inauthentic or paid, and expressing personal dislike or distrust of them even while acknowledging their broad reach

*“I hate social influencers. [...] Now, they are impactful. And they have a great following. [...] From an advertising business perspective, I think they work. Personally I hate influencers.” – Patient #8*

Some patients also suggested dating apps as promising platforms for PrEP promotion. They highlighted that these apps are widely used in LGBTQ+ communities and have already been effective in delivering HIV prevention resources and services. One specifically noted that dating apps were how they found out about a PrEP telehealth service they used to access PrEP.

*“Especially on different dating apps and social apps like [online dating apps]and things like that that are more targeted to the LGBT community, I think are good spots to kind of think about that and just good education in general about HIV prevention.” – Patient #6*

#### Theme 5: Word-of-mouth.

Most participants believed that recommendations from friends or peers would strongly influence their willingness to adopt a PrEP support tool. They noted that personal endorsements build trust, especially when based on positive experiences, and could make them more likely to view the service as credible and worth trying. For many, friends’ endorsements were seen as more persuasive than advertising.

*“If my friends told me about it, then definitely. I would, check it out. As a matter of fact, I think I believe it was a friend of ours that told us about [a PrEP telehealth service]. So yes, if it was from a friend, I would put it this way: I would much, much, much, much be more inclined to believe my friend.” – Patient #4*

However, a few were more skeptical of friends’ recommendations. Some felt that a single friend’s recommendation might not be enough to influence them unless echoed by multiple peers or discussed in a meaningful conversation. Others doubted that friends’ recommendations would be persuasive at all, citing privacy concerns, lack of trust in peers, or the likelihood of forgetting casual mentions without a clear incentive to act.

#### Theme 6: Outreach from health insurers.

No participants organically raised promotion through their insurance providers as a promising channel for promotion, but when asked about it, they had mixed reactions. Some noted that they already receive regular communication from their insurance companies and could imagine engaging with a PrEP-related program if it came through that channel.

Others expressed skepticism about insurance companies but still said they might be open to a program if it came from their provider. These participants acknowledged they tend to ignore insurance messages or questioned the companies’ motives, but still suggested they might look into it. For instance:

*“From my insurance? Yeah, I mean, I probably, you know, in the back of my head, I’d probably be like, okay, there’s probably some other kind of financial motive behind this, but sure, I’ll check it out.” – Patient #4*

Still other patients were much less trusting of insurers and said they would be unlikely to engage at all. These participants were concerned about financial motives, misuse of personal information, or simply preferred that insurers not be involved. As one patient described:

*“I don’t want my health insurance company to be involved in this. I think that health insurance companies are corporate America, make a buck… that would make me a little bit uncomfortable. … I don’t know if they’re going to use this information to increase my premium.” – Patient #8*

### Patient survey results

Survey responses showed that patients had a similarly positive view of the concept digital PrEP support program. Although we did not assess willingness or intention to adopt the product directly, patients reported similarly high acceptability (average item *M* = 4.25, *SD* = 0.91, Range = 2.25-5), feasibility (average item *M* = 4.81, *SD* = 0.35, Range = 4–5), and appropriateness (average item *M* = 3.84, *SD* = 0.92, Range = 2–5). Although these average ratings of both acceptability and appropriateness were slightly lower than that of clinic executives, they still reflect an overall average rating of “agree” across all constructs. Table 6 shows patients’ ratings of various potential dissemination strategies, in terms of how likely they would be to *notice* information presented and how likely each might be to encourage them to *sign up* (adopt) the tool. Strategies are sorted in terms of which were rated most highly across both dimensions with the lowest variability. Among the most highly-rated strategies were having a provider mention it to them during a visit, getting a message about the tool in their patient portals, seeing information about it in a post on social media, having clinic staff other than their providers mention it to them, and seeing their providers wear a button or sticker with information about the product on it. Several other strategies were rated less highly, but were still viewed as potentially effective, including digital and physical signage in clinics, information about the product included in a clinic newsletter, and messages (ideally letters) sent to them by their health insurance company. Table 7 shows participants’ ratings of various barriers to adoption they might have with respect to signing up for a similar PrEP adherence support program. Low perceived need for the program was the most highly rated barrier, followed by concerns about receiving more text messages, and skepticism about the benefits of the tool. However, it is important to highlight that none of the barriers assessed were rated as reasons participants would elect not to sign up for the program - the highest-rated item was slightly lower than ‘neutral,’ meaning that participants did not believe it would be an important reason for them to decide not to adopt it.

**Table 6:** Patients’ ratings of various dissemination strategies by how likely they would be to notice them and how likely they would be to adopt a digital PrEP support tool.

Strategy	*How likely to* notice*?*		*How likely to* sign up*?*		*Average rating*	
	*M*	*SD*	*M*	*SD*	*M*	*SD*
Your provider mentions it to you during a visit with you	5.00	0.00	4.88	0.35	4.94	0.18
A message in your patient portal from your clinic	4.50	0.76	4.38	0.92	4.44	0.84
A post on social media	4.38	0.52	4.00	1.07	4.19	0.79
Clinic staff (other than your provider) mentions it to you when you come to the clinic for a visit	4.13	1.25	4.25	1.16	4.19	1.21
Your provider is wearing a button or sticker with the product’s name on it	4.25	1.16	4.00	1.31	4.13	1.24
An ad on the TV in the clinic where you get your PrEP	4.13	0.83	3.63	1.41	3.88	1.12
A flyer posted in the clinic where you get your PrEP	4.00	1.31	3.75	1.39	3.88	1.35
A headline about it in your clinic’s email newsletter	3.75	1.58	3.88	1.64	3.81	1.61
A letter from your health insurance company	4.00	1.41	3.50	1.85	3.75	1.63
An ad on TV or a streaming service	4.13	0.83	3.25	1.49	3.69	1.16
An email from your health insurance company	3.75	1.58	3.13	1.89	3.44	1.73
A phone call from your health insurance company	3.50	1.51	2.88	1.73	3.19	1.62
A flyer posted around town	2.88	1.13	2.88	1.46	2.88	1.29
A business card placed on tables in the waiting areas of the clinic where you get your PrEP	3.00	1.20	2.75	1.39	2.88	1.29

**Table 7:** Summary of clinic executive and patient themes by dissemination strategy.

Type	In-clinic Strategies	Out-of-clinic Strategies
**Clinic Executives**	Theme 1: Direct Provider-to-Patient Recommendation, Staff Follow-up	Theme 4: Digital Marketing
	Theme 2: In-Clinic Physical Signage, with QR Codes	
	Theme 3: Post-visit Communications - Patient Portal Messages, Appointment Summaries, Emails, Texts	
**Patients**	Theme 1: Direct Provider-to-Patient Recommendation	Theme 4: Digital Marketing
	Theme 2: Post-visit Communications - Patient Portal Messages, Appointment Summaries, Emails	Theme 5: Word-of-Mouth
	Theme 3: In-clinic Physical & Digital Signage, Handouts	Theme 6: Outreach from Health Insurers

## Discussion

The goal of this study was to identify strategies that might be most effective for disseminating digital health products in clinical settings using a conceptual digital PrEP support tool as a concrete case for eliciting perspectives from both PrEP clinic executives and patients. Our goal was not to evaluate the conceptual tool itself, but to use it as a realistic anchor for discussions about dissemination approaches. This study adds to the literature by surfacing the broadest possible range of potential dissemination strategies, clarifying why executives and patients believe they might succeed or fail, and uncovering each group’s priorities and reservations about the product. Understanding these perspectives highlights which strategies are most likely to resonate with patients and fit within clinic workflows. This provides a stakeholder-informed foundation for campaign design that can be tailored to address specific priorities and concerns. It also serves as a starting point for formally testing and refining strategies in future studies to build more effective, evidence-based dissemination approaches.

### Provider engagement as the centerpiece of clinic-focused dissemination campaigns

Across both groups, the most consistent finding was the strong belief that direct provider-to-patient recommendations could be a particularly effective strategy. Established PrEP patients emphasized high levels of trust in their physicians, noting that if a provider takes the time to mention something, it is important. Executives echoed this, describing provider endorsement as uniquely authoritative compared to recommendations from other clinic staff. These findings align closely with prior work suggesting that face-to-face provider referrals are among the most influential facilitators of digital health adoption, often yielding stronger engagement than more diffuse marketing efforts [11,15].

At the same time, executives acknowledged providers’ time constraints, raising an important tension: while providers’ referrals are indispensable, they are not easily sustained in already overburdened workflows. Our qualitative findings suggest several ways this challenge might be addressed. One promising approach involves providers offering a brief mention of the tool, followed by more in-depth follow-up from other staff (e.g., nurses, medical assistants, navigators). Consistent with past research [8], executives also pointed to appointing a local “champion,” a staff member with dedicated time to promote the tool in various ways among clinicians and patients, as perhaps the single most effective way to ensure providers consistently recommend it. These champions could be charged with implementing many of the strategies that were rated most highly by executives in terms of those most likely to encourage providers to recommend the tool, such as leading discussions about it in clinic staff meetings, conducting dedicated demonstrations, or just discussing it frequently with colleagues. Together, these results underscore that provider referral should form the foundation of dissemination efforts, but encouraging providers to do so consistently would require a combination of strategies, such as staff engagement, ongoing reminders, and visible cues, that could be most effectively coordinated by a local champion.

### Clinic staff and other in-clinic promotion strategies

Both executives and patients also identified non-provider staff as important players in promoting new services. Established PrEP patients described trust in a wide range of clinical and non-clinical staff, and executives saw opportunities for staff to reinforce provider messages. These findings highlight the value of a “whole clinic” approach in which multiple staff members are involved in promotion and potentially contribute to different parts of the process. For example, providers may mention the tool and provide handouts about it during patient visits, and then support staff (e.g., nurses, medical assistants, patient navigators) can provide more information, training (if necessary) and answer any questions. Both groups also considered physical or digital signage in clinics as useful complementary strategies, particularly when paired with QR codes or patient portal notifications. Importantly, the rationale for these strategies was not that they could stand alone, but rather that they could save provider time while still ensuring patients had credible, clinic-associated sources of information.

These findings extend previous research by showing that while patients and executives did see value in signage, handouts, and clinic-based digital messages, they viewed these as helpful complements rather than stand-alone solutions. Consistent with prior studies that have noted such strategies are important but could be insufficient on their own [15], our results suggest they may be more effective when paired with other clinic-based efforts that establish credibility, such as provider or staff recommendations. In other words, these approaches appear most promising when they reinforce interpersonal promotion, rather than serve as the sole means of dissemination.

### Differing views on external marketing and payer outreach

Where executives and patients diverged most sharply was in their views of broader marketing approaches. Executives were relatively enthusiastic about social media promotion, particularly as a way to reach younger patients. In contrast, many patients disagreed. Although some noted that they pay attention to ads or influencer posts on social media, many doubted their authenticity or relevance. A few suggested dating apps as more promising venues, reflecting their established role in HIV prevention outreach. While social media promotion may reliably drive a higher absolute number of people to a product, prior research suggests that many of those reached will engage only minimally [11] and that a substantial portion of advertising budgets may be spent reaching people who are not good candidates for the tool, such as those not currently using or considering PrEP. Together, these findings suggest that while social media advertising may help generate broad awareness, its efficiency for promoting adoption of digital health tools focused on particular clinical populations (e.g., patients who take PrEP) could be limited. Campaigns may achieve greater impact by prioritizing clinic-based dissemination strategies that target individuals already engaged in PrEP care, using social media and other broad marketing methods as secondary approaches to supplement these measures.

We examined participants’ views of insurer outreach because health insurers, like clinics, are in a position to identify members who have been prescribed PrEP and could in principle contact them directly about a support tool. There is precedent for this approach. For example, Blue Cross Blue Shield of Massachusetts has used targeted portal messages to members with or at risk for diabetes to promote digital health products such as Livongo and Virta Health [27]. In our study, patients’ reactions to insurer-based outreach were mixed. Some indicated they might respond to messages that mentioned PrEP explicitly, but many expressed skepticism, citing low trust in insurers’ motives, discomfort with data use, or a tendency to ignore insurer communications altogether. These findings suggest that while insurers may be able to reach the appropriate population at scale, their impact on adoption is uncertain. Insurer outreach may be most useful as a supplementary strategy, and future work should test whether carefully framed messages can overcome concerns about credibility and trust.

### Informing the content of dissemination materials

We assessed which characteristics of the proposed PrEP support tool executives viewed as most important, not to guide the design of the tool itself (preferences for features like low cost, ease of use, and minimal workflow disruption are already well-established [28–31]), but to inform what elements might be emphasized in promotional content. Executives in this study placed the highest importance on ease of patient sign-up, strong evidence of effectiveness, low cost, and ease of referral for providers, whereas factors such as testimonials from other patients or providers were seen as less important. Since advertising space is limited, knowing which attributes executives value most can help ensure that marketing materials highlight the features most likely to capture their attention and motivate promotion within clinics. Similarly, we asked patients about barriers that might shape their decision to adopt a similar tool, in order to identify what messages would be most useful to address directly. The most commonly endorsed barrier was a low perceived need, a reflection of their high adherence and longer PrEP use duration. This suggests that promotional content should explicitly counteract this concern—for instance, by including messaging such as *‘Even if you’ve been on PrEP for years, this tool can help you build a routine that lasts.’* By tailoring content in this way to reflect the specific priorities and reservations of both executives and patients, dissemination campaigns can more directly address the factors most likely to shape adoption.

## Limitations

Although this study had many strengths, several limitations are important to note. Our sample was small and patients largely reflected a relatively privileged set of more established PrEP patients. Preferred dissemination strategies, and the trust patients have in them, may differ substantially among less-privileged patients. In this study, we elected to focus on men and transgender women, which may limit generalizability. This study also focused on stakeholder-perceived strategies that may be effective, but did not examine variation by race and ethnicity, which may constrain the broader applicability of the findings. Frontline providers and clinicians were not explicitly recruited for this study. While we did not collect data on clinical background for this study, clinic executives were chosen because they actively participate in executive decision making for clinics and direct patient care. Many clinic executives are frontline providers or former providers themselves. Patient participants had unusually long experience with PrEP, high adherence, and low difficulty taking PrEP which may have tempered enthusiasm for the concept product and led them to downplay potential benefits. The established PrEP patients also limit generalizability among younger, less adherence populations on PrEP. For executives, our survey asked which strategies might be effective, but not which they would be most likely to notice or act on. It could be that, like patients, certain strategies might be more effective for increasing awareness of products among providers and others more effective in encouraging buy-in. Finally, although our study identified a wide range of possible strategies that key stakeholders believed could be effective, it did not explicitly test their relative effectiveness on actual adoption.

## Conclusion

This study contributes to the dissemination literature by identifying a broad set of strategies for promoting digital health products in clinics and clarifying why stakeholders believe particular strategies matter. Both executives and patients highlighted provider endorsement as the most promising approach for driving adoption, with complementary roles for other staff and in-clinic promotion. External advertising and insurer outreach may also be effective, but some participants had important reservations that could limit their effectiveness. By centering stakeholder perspectives, these findings provide a playbook for designing dissemination campaigns that are both credible and feasible, ultimately helping digital health products overcome the research-to-practice gap and realize their potential in supporting PrEP adherence. Future research should build on these findings by systematically evaluating dissemination strategies identified here. It should feature more diverse populations and larger sample sizes, including an examination of how race and ethnicity might moderate strategies and should include an analysis with digital health tools geared towards varied health outcomes and patient communities. Subsequent studies should also examine validity of novel survey measures and test combinations of approaches that balance feasibility for clinics with persuasiveness for patients. Doing so could help ensure that campaigns achieve meaningful real-world adoption and sustained use of evidence-based digital health tools.

## Supporting information

S1 Appendix A list of southern US states. (DOCX)

S2 Appendix Mockups of intervention sign up website on desktop and mobile. (DOCX)

S3 Appendix Quantitative survey measures. (DOCX)

## References

[1] HIV.gov. What is ‘Ending the HIV Epidemic: A Plan for America’?. 2019. https://www.hiv.gov/ending-hiv-epidemic

[2] Centers for Disease Control and Prevention. Fast Facts: HIV in the United States. 2024. https://www.cdc.gov/hiv/data-research/facts-stats/index.html

[3] Skarbinski J, Rosenberg E, Paz-Bailey G, Hall HI, Rose CE, Viall AH, et al. Human immunodeficiency virus transmission at each step of the care continuum in the United States. JAMA Intern Med. 2015;175(4):588–96. doi: 10.1001/jamainternmed.2014.8180 25706928

[4] Prevention CfDCa. PrEP for HIV Prevention in the U.S. 2023.

[5] Bedimo RJ, Jodlowski T. PrEParing for the End of the HIV Epidemic-A Public Health Imperative. JAMA Netw Open. 2023;6(8):e2330195. doi: 10.1001/jamanetworkopen.2023.30195 37606931

[6] Wray TB, Chan PA, Klausner JD, Ward LM, Ocean EMS. Gay, Bisexual, and Other Men Who Have Sex With Men Who Are Not on Oral PrEP may be Less Interested in Available Injectable Products than in Oral PrEP: Examining Individual-Level Determinants of Interest and Barriers Across Products. AIDS Behav. 2022;26(11):3794–805. doi: 10.1007/s10461-022-03708-3 35583574 PMC9912751

[7] US Preventive Services Task Force. HIV Prevention: Digital Health Interventions to Improve Adherence to HIV Pre-Exposure Prophylaxis. 2021. https://www.thecommunityguide.org/findings/hiv-prevention-digital-health-interventions-improve-adherence-hiv-pre-exposure-prophylaxis.html

[8] Borges do Nascimento IJ, Abdulazeem H, Vasanthan LT, Martinez EZ, Zucoloto ML, Østengaard L, et al. Barriers and facilitators to utilizing digital health technologies by healthcare professionals. NPJ Digit Med. 2023;6(1):161. doi: 10.1038/s41746-023-00899-4 37723240 PMC10507089

[9] Moungui HC, Nana-Djeunga HC, Anyiang CF, Cano M, Ruiz Postigo JA, Carrion C. Dissemination Strategies for mHealth Apps: Systematic Review. JMIR Mhealth Uhealth. 2024;12:e50293. doi: 10.2196/50293 38180796 PMC10799285

[10] Kvedarienė V, Biliute G, Didziokaitė G, Kavaliukaite L, Savonyte A, Rudzikaite-Fergize G, et al. Mobile health app for monitoring allergic rhinitis and asthma in real life in Lithuanian MASK-air users. Clin Transl Allergy. 2022;12(9):e12192. doi: 10.1002/clt2.12192 36178186 PMC9510653

[11] Hui CY, McKinstry B, Walton R, Pinnock H. Strategies to promote adoption and usage of an application to support asthma self-management: a qualitative observational study. BMJ Health Care Inform. 2018;25(4):243–53. doi: 10.14236/jhi.v25i4.1056 30672405

[12] Roberts AL, Potts HW, Koutoukidis DA, Smith L, Fisher A. Breast, Prostate, and Colorectal Cancer Survivors’ Experiences of Using Publicly Available Physical Activity Mobile Apps: Qualitative Study. JMIR Mhealth Uhealth. 2019;7(1):e10918. doi: 10.2196/10918 30609982 PMC6329432

[13] Rajani NB, Mastellos N, Filippidis FT. Impact of Gamification on the Self-Efficacy and Motivation to Quit of Smokers: Observational Study of Two Gamified Smoking Cessation Mobile Apps. JMIR Serious Games. 2021;9(2):e27290. doi: 10.2196/27290 33904824 PMC8114162

[14] Buss VH, Varnfield M, Harris M, Barr M. Remotely Conducted App-Based Intervention for Cardiovascular Disease and Diabetes Risk Awareness and Prevention: Single-Group Feasibility Trial. JMIR Hum Factors. 2022;9(3):e38469. doi: 10.2196/38469 35776504 PMC9288098

[15] Mair JL, Hashim J, Thai L, Tai ES, Ryan JC, Kowatsch T, et al. Understanding and overcoming barriers to digital health adoption: a patient and public involvement study. Transl Behav Med. 2025;15(1):ibaf010. doi: 10.1093/tbm/ibaf010 40167046 PMC11959363

[16] Shea CM. A conceptual model to guide research on the activities and effects of innovation champions. Implement Res Pract. 2021;2:10.1177/2633489521990443. doi: 10.1177/2633489521990443 34541541 PMC8445003

[17] Yang L, Brown-Johnson CG, Miller-Kuhlmann R, Kling SMR, Saliba-Gustafsson EA, Shaw JG, et al. Accelerated launch of video visits in ambulatory neurology during COVID-19: Key lessons from the Stanford experience. Neurology. 2020;95(7):305–11. doi: 10.1212/WNL.0000000000010015 32611634

[18] Pettersen S, Eide H, Berg A. The role of champions in the implementation of technology in healthcare services: a systematic mixed studies review. BMC Health Serv Res. 2024;24(1):456. doi: 10.1186/s12913-024-10867-7 38605304 PMC11007964

[19] Spicehandler R, Zucker J, Yumori C, Adan M, Carnevale C, Theodore D, et al. Get2PrEP: An EMR lab comment increased safe sex counseling but not PrEP services at a large urban academic medical center in Northern Manhattan. Sexually Transmitted Diseases. 2022. doi: 10.1097/OLQ.0000000000001682 PMC9481682 35921642

[20] Moungui HC, Iyawa PT, Nana-Djeunga H, Ruiz-Postigo JA, Carrion C. Implementation of a marketing plan for the dissemination of the WHO SkinNTDs app in Cameroon. PLoS One. 2025;20(9):e0333295. doi: 10.1371/journal.pone.0333295 40997058 PMC12463274

[21] Vis C, Schuurmans J, Aouizerate B, Atipei Craggs M, Batterham P, Bührmann L, et al. Effectiveness of Self-guided Tailored Implementation Strategies in Integrating and Embedding Internet-Based Cognitive Behavioral Therapy in Routine Mental Health Care: Results of a Multicenter Stepped-Wedge Cluster Randomized Trial. J Med Internet Res. 2023;25:e41532. doi: 10.2196/41532 36735287 PMC9938445

[22] Brownson RC, Eyler AA, Harris JK, Moore JB, Tabak RG. Getting the Word Out: New Approaches for Disseminating Public Health Science. J Public Health Manag Pract. 2018;24(2):102–11. doi: 10.1097/PHH.0000000000000673 28885319 PMC5794246

[23] Liu AY, Vittinghoff E, von Felten P, Rivet Amico K, Anderson PL, Lester R, et al. Randomized Controlled Trial of a Mobile Health Intervention to Promote Retention and Adherence to Preexposure Prophylaxis Among Young People at Risk for Human Immunodeficiency Virus: The EPIC Study. Clin Infect Dis. 2019;68(12):2010–7. doi: 10.1093/cid/ciy810 30239620 PMC6541706

[24] Colson PW, Franks J, Wu Y, Winterhalter FS, Knox J, Ortega H, et al. Adherence to Pre-exposure Prophylaxis in Black Men Who Have Sex with Men and Transgender Women in a Community Setting in Harlem, NY. AIDS Behav. 2020;24(12):3436–55. doi: 10.1007/s10461-020-02901-6 32385678

[25] Proctor E, Silmere H, Raghavan R, Hovmand P, Aarons G, Bunger A. Outcomes for implementation research: conceptual distinctions, measurement challenges, and research agenda. Administration and Policy in Mental Health and Mental Health Services Research. 2011;38(2):65–76. 20957426 10.1007/s10488-010-0319-7 PMC3068522

[26] Weiner BJ, Lewis CC, Stanick C, Powell BJ, Dorsey CN, Clary AS, et al. Psychometric assessment of three newly developed implementation outcome measures. Implement Sci. 2017;12(1):108. doi: 10.1186/s13012-017-0635-3 28851459 PMC5576104

[27] Massachusetts BCaBSo. Personalized care & support for members diagnosed with diabetes. 2023. https://provider.bluecrossma.com/ProviderHome/portal/home/news/news/clinical-and-pharmacy/all%20networks/personalized%20care%20and%20support%20for%20members%20diagnosed%20with%20diabetes%20(secure%20and%20non-secure)

[28] Zhou L, Parmanto B. User Preferences for Privacy Protection Methods in Mobile Health Apps: A Mixed-Methods Study. Int J Telerehabil. 2020;12(2):13–26. doi: 10.5195/ijt.2020.6319 33520091 PMC7757650

[29] Peng W, Kanthawala S, Yuan S, Hussain SA. A qualitative study of user perceptions of mobile health apps. BMC Public Health. 2016;16(1):1158. doi: 10.1186/s12889-016-3808-0 27842533 PMC5109835

[30] Ramanathan N, Swendeman D, Comulada WS, Estrin D, Rotheram-Borus MJ. Identifying preferences for mobile health applications for self-monitoring and self-management: focus group findings from HIV-positive persons and young mothers. Int J Med Inform. 2013;82(4):e38-46. doi: 10.1016/j.ijmedinf.2012.05.009 22704234 PMC3473096

[31] Yoon S, Goh H, Nadarajan GD, Sung S, Teo I, Lee J, et al. Perceptions of Mobile Health Apps and Features to Support Psychosocial Well-being Among Frontline Health Care Workers Involved in the COVID-19 Pandemic Response: Qualitative Study. J Med Internet Res. 2021;23(5):e26282. doi: 10.2196/26282 33979296 PMC8168635

