# Supplementary material for: Identifying dissemination strategies for promoting adoption of digital health interventions in clinical settings: A convergent parallel study on text message support for HIV pre-exposure prophylaxis (PrEP) adherence
Source: PLOS Digit Health. 2026 Jul 13;5(7):e0001117. doi: 10.1371/journal.pdig.0001117 (PMC13362131; doi:10.1371/journal.pdig.0001117)
Supplement: S1 Appendix — (DOCX) [file pdig.0001117.s001.docx]

S1 Appendix: A list of Southern US states

Alabama, Arkansas, Delaware, District of Columbia, Florida, Georgia, Kentucky, Louisiana, Maryland, Mississippi, North Carolina, South Carolina, Tennessee, Texas, Virginia, or West Virginia
