# Supplementary figures and images for: Identifying dissemination strategies for promoting adoption of digital health interventions in clinical settings: A convergent parallel study on text message support for HIV pre-exposure prophylaxis (PrEP) adherence
Source: PLOS Digit Health. 2026 Jul 13;5(7):e0001117. doi: 10.1371/journal.pdig.0001117 (PMC13362131; doi:10.1371/journal.pdig.0001117)

S2 Appendix: Mockups of intervention sign up website on desktop and mobile.


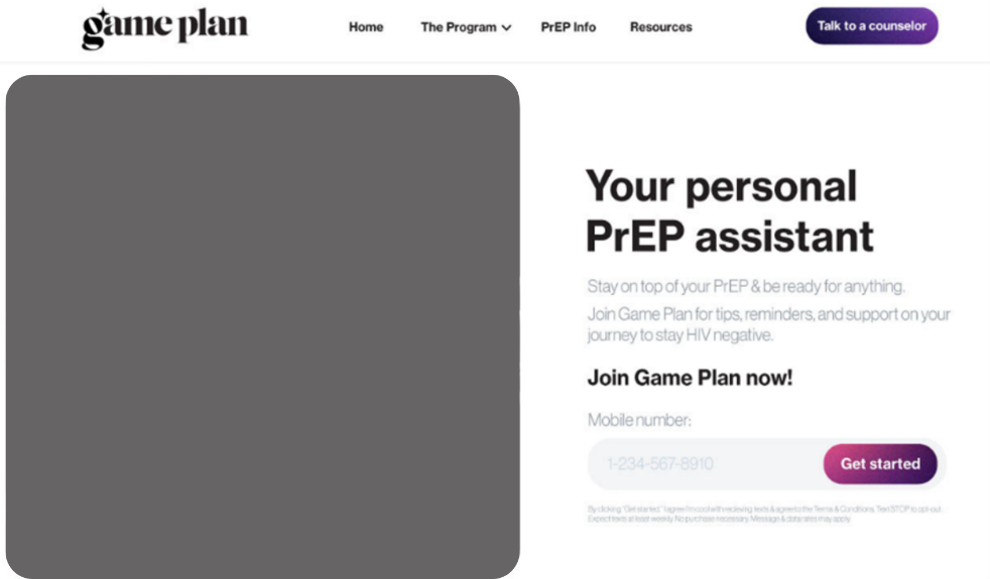

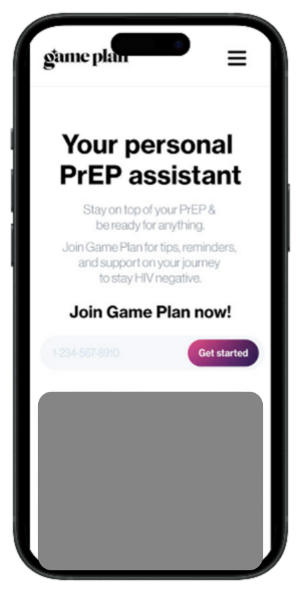

Supplement: S2 Appendix — (DOCX) [file pdig.0001117.s002.docx]
