# Supplementary material for: Identifying dissemination strategies for promoting adoption of digital health interventions in clinical settings: A convergent parallel study on text message support for HIV pre-exposure prophylaxis (PrEP) adherence
Source: PLOS Digit Health. 2026 Jul 13;5(7):e0001117. doi: 10.1371/journal.pdig.0001117 (PMC13362131; doi:10.1371/journal.pdig.0001117)
Supplement: S3 Appendix — (DOCX) [file pdig.0001117.s003.docx]

S3 Appendix: Quantitative Survey Measures

Clinic executive survey measures:

| The following items represent different ways you might hear about Game Plan. For each item, rate whether you think you would be likely to take notice of Game Plan based on hearing about it that way. | Not likely at all (1) | Not very likely (2) | Neutral (3) | Somewhat likely (4) | Very likely (5) |
| --- | --- | --- | --- | --- | --- |
| 1. A flyer posted in the clinic where you work |  |  |  |  |  |
| 2. A business card placed on tables in common areas (e.g., conference room) at the clinic where you work |  |  |  |  |  |
| 3. An ad on a TV or screen in the clinic where you work |  |  |  |  |  |
| 4. A headline about it in the email newsletter of the clinic where you work |  |  |  |  |  |
| 5. A message to you in your electronic health records system that the clinic you work at uses |  |  |  |  |  |
| 6. Someone presenting about it in your regular staff meeting at the clinic where you work |  |  |  |  |  |
| 7. Someone presenting about it in a special presentation at the clinic where you work |  |  |  |  |  |
| 8. A colleague who works with you at your clinic directly mentioning it to you |  |  |  |  |  |
| 9. A colleague who works with you at your clinic handing out or wearing promotional materials about it, like buttons, pens, or information cards |  |  |  |  |  |
| 10. A text message sent directly to you |  |  |  |  |  |

| Below are several factors that might affect your willingness to recommend that your patients use a program like Game Plan. Please rate each item according to how important it would be to your decision to recommend or promote a program like this. | Not at all important (1) | Not very important (2) | Neutral (3) | Somewhat important (4) | Very important (5) |
| --- | --- | --- | --- | --- | --- |
| The research evidence showing that it encourages more consistent use of PrEP (1) |  |  |  |  |  |
| How easy it is for patients to sign up for it (2) |  |  |  |  |  |
| Its security (3) |  |  |  |  |  |
| Its cost (4) |  |  |  |  |  |
| Whether it addresses a specific topic that contributes to poor adherence, like stigma or risk perceptions (5) |  |  |  |  |  |
| How easy it is for you to refer people to it (6) |  |  |  |  |  |
| Its promotional materials (website, ads, flyers) look professional (7) |  |  |  |  |  |
| Testimonials from patients saying it was helpful for them (8) |  |  |  |  |  |
| Testimonials from other providers saying it helped their patients (9) |  |  |  |  |  |

Patient survey measures:

| The following items represent different ways you might hear about Game Plan. For each item, rate whether you think you would be likely to *notice* Game Plan based on hearing about it that way.  *AND*  The following items represent different ways you might hear about Game Plan. For each item, rate whether you think you would be likely to *sign up* for Game Plan based on hearing about it that way. | Not likely at all (1) | Not very likely (2) | Neutral (3) | Somewhat likely (4) | Very likely (5) |
| --- | --- | --- | --- | --- | --- |
| 1. A flyer posted in the clinic where you get your PrEP |  |  |  |  |  |
| 2. A business card placed on tables in the waiting room of the clinic where you get your PrEP |  |  |  |  |  |
| 3. An ad on the TV in the clinic where you get your PrEP |  |  |  |  |  |
| 4. A flyer posted around town |  |  |  |  |  |
| 5. An ad on TV or a streaming service |  |  |  |  |  |
| 6. A post on social media |  |  |  |  |  |
| 7. A headline about it in your clinic's email newsletter |  |  |  |  |  |
| 8. A message in your patient portal from your clinic |  |  |  |  |  |
| 9. An email from your health insurance company |  |  |  |  |  |
| 10. A letter from your health insurance company |  |  |  |  |  |
| 11. A phone call from your insurance company |  |  |  |  |  |
| 12. Your provider mentions it to you during a visit with you |  |  |  |  |  |
| 13. Your provider is wearing a button with [product's name] on it |  |  |  |  |  |
| 14. Clinic staff (other than your provider) mentions it to you when you come to the clinic for a visit |  |  |  |  |  |

| The following items ask you about reasons you might elect not to sign up for Game Plan. Rate each item according to whether that reason is relevant to you. | Not at all a reason why I wouldn't sign up (1) | Not really a reason why I wouldn't sign up (2) | Neutral (3) | Probably a reason why I wouldn't sign up (4) | Very much a reason why I wouldn't sign up (5) |
| --- | --- | --- | --- | --- | --- |
| 1. I don't have a phone or access to free text messaging |  |  |  |  |  |
| 2. It doesn't sound interesting to me |  |  |  |  |  |
| 3. I don't think I need it |  |  |  |  |  |
| 4. It seems like a scam |  |  |  |  |  |
| 5. I don't want to go through the effort of signing up |  |  |  |  |  |
| 6. I don't want to get tons more text messages |  |  |  |  |  |
| 7. I don't see how it would benefit me |  |  |  |  |  |
| 8. I don't think it would help me take my PrEP regularly |  |  |  |  |  |
| 9. No one else I know has used it |  |  |  |  |  |
